# Use of daily Internet search query data improves real-time projections of influenza epidemics

**DOI:** 10.1098/rsif.2018.0220

**Published:** 2018-10-10

**Authors:** Christoph Zimmer, Sequoia I. Leuba, Reza Yaesoubi, Ted Cohen

**Affiliations:** 1 Epidemiology of Microbial Diseases, Yale School of Public Health, New Haven, CT, USA; 2 Health Policy and Management, Yale School of Public Health, New Haven, CT, USA; 3 Bosch Center for Artificial Intelligence, Robert Bosch GmbH, Renningen, Germany

**Keywords:** influenza, transmission dynamics, forecasting, data resolution, Wikipedia

## Abstract

Seasonal influenza causes millions of illnesses and tens of thousands of deaths per year in the USA alone. While the morbidity and mortality associated with influenza is substantial each year, the timing and magnitude of epidemics are highly variable which complicates efforts to anticipate demands on the healthcare system. Better methods to forecast influenza activity would help policymakers anticipate such stressors. The US Centers for Disease Control and Prevention (CDC) has recognized the importance of improving influenza forecasting and hosts an annual challenge for predicting influenza-like illness (ILI) activity in the USA. The CDC data serve as the reference for ILI in the USA, but this information is aggregated by epidemiological week and reported after a one-week delay (and may be subject to correction even after this reporting lag). Therefore, there has been substantial interest in whether real-time Internet search data, such as Google, Twitter or Wikipedia could be used to improve influenza forecasting. In this study, we combine a previously developed calibration and prediction framework with an established humidity-based transmission dynamic model to forecast influenza. We then compare predictions based on only CDC ILI data with predictions that leverage the earlier availability and finer temporal resolution of Wikipedia search data. We find that both the earlier availability and the finer temporal resolution are important for increasing forecasting performance. Using daily Wikipedia search data leads to a marked improvement in prediction performance compared to weekly data especially for a three- to four-week forecasting horizon.

## 1. Introduction

Seasonal influenza remains an important infectious cause of morbidity and mortality [1,2]. In the USA alone, estimates of annual incidence range from 9.2 million to 35.6 million cases, resulting in 140 000 to 710 000 hospitalizations and 12 000 to 56 000 deaths [3].

Efforts to improve *nowcasts* and short-term predictions of influenza activity are motivated by the need to anticipate intensive care unit crowding and surges in vaccine demand. The US Centers for Disease Control and Prevention (CDC) recognize the importance of improving methods for assessing short-term predictions of influenza activity by hosting an annual influenza forecasting challenge [4]. Several data sources have been used to monitor the influenza activity in the USA and as calibration data for forecasting models. Data include both direct measures of influenza such as the official CDC influenza-like illness (ILI) data [5] and indirect measures such as Internet search queries. The validity of Internet search queries, which have included Google [6–12], Twitter [13,14] and Wikipedia [15,16], as proxy measures of influenza activity have been the subject of some debate [12]. However, the rapid availability, low cost of acquisition and potential to access Internet search query data at relatively high spatial resolution make these data sources attractive options for many teams participating in the annual CDC's influenza prediction challenge [17].

The approaches utilized for influenza prediction in the CDC challenge vary widely, with methods that range from compartmental dynamic transmission models [18–22] to non-mechanistic approaches [23,24]. While previously described models utilize weekly aggregated data on influenza activity [16,18–20,23,24], we note that Internet search query data are often available at much higher temporal resolution, and we sought to understand whether leveraging daily search data can improve short-term predictions of influenza activity. Here we evaluate the comparative performance of models utilizing CDC ILI data to models which utilize Wikipedia data that despite being a more indirect measure of influenza are available more rapidly than ILI data and at finer temporal resolution.

## 2. Material and methods

Here we describe the availability of Wikipedia search data and demonstrate its utility as a proxy measure of influenza activity, introduce a mechanistic model of influenza transmission and define our framework for model calibration and prediction.

### 2.1. Wikipedia data

#### 2.1.1. Weekly aggregated search data

The CDC defines ILI as a ‘fever (temperature of 100°F [37.8°C] or greater) and a cough and/or a sore throat without a KNOWN cause other than influenza’ [25] and per cent ILI is the percentage of the total patient visits related to an ILI.

The correspondence of Wikipedia search data [26] and CDC ILI data has been previously demonstrated by others [15]. In more recent work, Wikipedia data have been used as the basis for influenza forecasting in the USA. Hickmann *et al.* [16] developed a linear regression to map searches of selected Wikipedia articles to CDC ILI data. This model calculates a CDC ILI estimate based on the previous week's CDC ILI, *ILI*_−1_ and current week's numbers of Wikipedia searches for relevant Wikipedia articles. The most predictive article names included ‘Human Flu’, ‘Influenza’, ‘Influenza A virus’, ‘Influenza B virus’ and ‘Oseltamivir’. Their regression reads as2.1
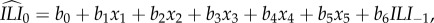
where 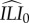 is the current ILI estimate based on Wikipedia data, *x*_*i*_ is the ratio of the number of visits of these articles to the total number of visited pages, and the regression coefficients are *b*_0_ = 0.0063, *b*_1_ = 17517.3, *b*_2_ = 3206.1, *b*_3_ = 41258.9, *b*_4_=− 71428.7, *b*_5_ = −17410.9 and *b*_6_ = 0.955 [16]. In equation (2.1), the unit of ILI is ‘% influenza among total visits’, and the unit of *x*_1_, …, *x*_5_ is ‘# page searches/# total searches’, the unit of *b*_1_, …, *b*_5_ consequently is ‘% influenza among total visits/(# page searches/# total searches)’, the unit of *b*_0_ ‘% influenza among total visits’ and *b*_6_ is dimensionless.

**Figure 1.**
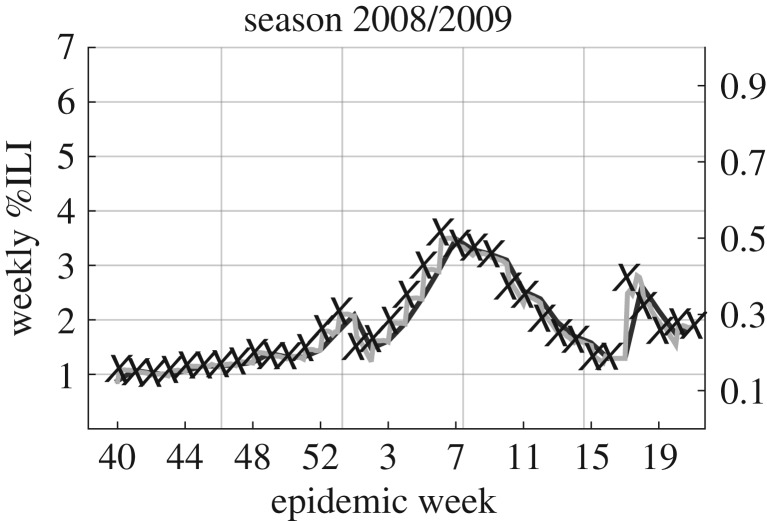
Wikipedia search data provide good fits to CDC ILI data. The CDC ILI data from the season 2008/2009 are in black crosses, estimates obtained from Wikipedia searches with weekly aggregation are in dark grey and estimates obtained from Wikipedia searches in daily resolution are in light grey.

Even though this model is purely phenomenological, Hickmann *et al.* show that this model produces a good fit to CDC ILI data [16]. However, Hickmann *et al.* [16] also note that the regression coefficients should not be used to infer an importance of the predictors as page visits and ILI are not on the same scale. In figure 1, we visualize this fit for the 2008/2009 season (other years produce similarly good fits as shown in electronic supplementary material, figure A2).

#### 2.1.2. Daily search data

While previous work has used weekly aggregated Wikipedia search data, we note that the data are available at finer temporal resolution. We extend the formula that links weekly Wikipedia data to CDC ILI to create a daily ILI estimate (figure 1) using the coefficients determined in the previous regression:2.2
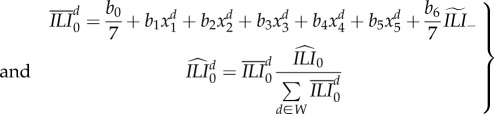
where *d* stands for the day number of the current week, *x*^*d*^_1_, …, *x*^*d*^_5_ are the daily page visit ratios and 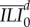 is the unnormalized daily ILI estimate based on Wikipedia, with 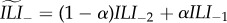. The set *W* consists of the days corresponding to the week of data collection. In equation (2.2), the unit of the daily ILI estimate, 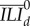, is ‘% influenza-related visits among total visits per day’, the unit of 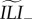 is as in equation (2.1) ‘% influenza among total visits (per week)’, the unit of *x*^*d*^_1_, …, *x*^*d*^_5_ is ‘# page searches/# total searches (per day)’ and consequently the unit of *b*_1_, …, *b*_5_ is ‘% influenza among total visits (per day)/(# page searches/# total searches (per day))’, the unit of *b*_0_ is ‘% influenza-related visits among total visits per day’ and *b*_6_ is dimensionless.

The normalization (second line of equation (2.2)) allows the daily Wikipedia data to sum up to the values of the weekly Wikipedia data. *ILI*_−1_ denotes the previous week's CDC's ILI and *ILI*_−2_, the CDC's ILI from two weeks ago. Therefore, 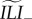 is a *α* weighted combination. The smoothing factor *α* can be set to

(a) *α* = 1 (no smoothing)
(b) *α* = *d*/7 (smoothing) or
(c)
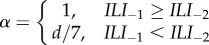
 (partial smoothing)

Partial smoothing is thus equal to no smoothing as long as the data trajectory is rising and equal to smoothing if the data trajectory is falling. The reason to introduce these different kinds of smoothing is the following: the daily estimate of the last day of a week depends only on the CDC's ILI of the beginning of the week, while 1 day later, the daily estimate of a first day of a week depends only on the CDC's ILI estimate of the next week. This sort of discontinuity can lead to less smooth behaviour as depicted in figure 1 and can be addressed by introducing the smoothing factor *α*. We will use the partial smoothing in the main text as it seems to have the best performance (electronic supplementary material, figure A6). If a data point, *x*^*d*^_*i*_, is missing (this happens for 8 days in epidemic weeks 16 and 17 of 2009 and for 4 days in December 2011), the last previous valid data point is used.

### 2.2. Computational model

A humidity-based susceptible–infected–recovered–susceptible (SIRS) influenza model has been developed by Shaman and colleagues [18,19]. The model includes the following transitions:2.3
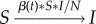
2.4
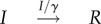
2.5
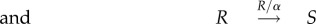
with an average duration of immunity *α*, a mean infectious period *γ*, and a transmission rate *β*(*t*) which is defined as *β*(*t*) = *R*_0_(*t*)/*γ* with an 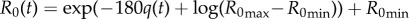 with a maximal and minimal daily reproductive number 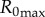 and 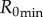, and a function *q* describing the absolute humidity (see electronic supplementary material, Humidity data). The effective reproductive number is *R*_eff_(*t*) = *S*(*t*)/*N* · *R*_0_(*t*).

Assuming constant population size *N*_pop_ = *S* + *I* + *R*, it holds that *R* = *N*_pop_ − *S* − *I*, and we can reduce the model by eliminating the third equation and replacing *R* by *N*_pop_−*S*− *I*. We summarize the state of the epidemic consisting of a continuous relaxation of the number of susceptibles, infected and recovered, as *ν* = (*ν*^(S)^, *ν*^(I)^, *ν*^(R)^). As the initial states are unknown, we treat them as additional model parameters and we summarize the parameters in one parameter vector *θ*, 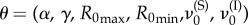.

The CDC ILI data (and the Wikipedia proxy) reflect the daily or weekly number of incident infections. To apply the MSS method, we need to specify how to map observations *y*_*i*_, e.g. daily or weekly number of incident infections, to states *ν*_*i*_ (the number of susceptibles *ν*^(S)^_*i*_, infected *ν*^(I)^_*i*_ and recovered *ν*^(R)^_*i*_). As in [27], we describe the new cases *y*_*i*_ in a time interval [*t*_*i*−1_, *t*_*i*_] as the difference between the number of infected at the end, *ν*^(I)^_*i*_, and number of infected at the beginning, *ν*^(I)^_*i*−1_, plus the number of recoveries, denoted by R*_i_. This number of new recoveries is not simply the difference in numbers of people in the *R* compartment between the beginning and end of the interval [*t*_*i*−1_, *t*_*i*_] as people might lose immunity and, hence, move from *R* to *S*. Therefore, we track the number of recoveries in each time interval [*t*_*i*−1_, *t*_*i*_] by introducing an artificial compartment R* of newly recovered people that is initialized with 0 at the start of each interval.

Our model does not differentiate between different influenza strains or non-influenza causes for ILI. We note that while this is a strong simplifying modelling assumption, it appears to work sufficiently well as demonstrated by [16] and also in our work.

The model is considered as a continuous time stochastic model. Forward simulation will be created by using Gillespie's stochastic simulation algorithm [28]. The calibration method will be introduced in the next section.

### 2.3. Calibration and prediction

Observations *y*_1_, …, *y*_*n*_ are recorded at time points *t*_1_, …, *t*_*n*_. The observations consist in this study of weekly or daily new ILI cases. We use an iterative procedure to update our knowledge at the time of each new observation. We use a prior distribution *π*_0_(*θ*) to represent our existing knowledge on the epidemic parameters before the first observation. We use flat uniform priors for the parameters 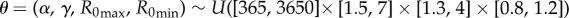 and the initial states *S*_0_, *I*_0_∼*U*([50 000,  70 000] × [10, 100]).

As each new observation *y*_*i*_ accumulates, we update our knowledge on the parameter *θ* by multiplying our prior with the probability of observing *y*_*i*_:2.6
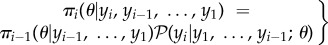
Note that the posterior at time *t*_*i*−1_, *π*_*i*−1_, also serves as the prior at time *t*_*i*_. For *i* = 1 we set *π*_0_(*θ* | *y*_0_) = *π*_0_(*θ*) as we do not have any observations at time *t*_0_. The two following subsections explain how we set up a suitable approximation for 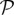 and how we propagate the distribution *π*_*i*_ through time.

#### 2.3.1. Likelihood approximation

We use the multiple shooting for stochastic systems (MSS) method to approximate 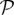. The MSS method is fast enough to be computationally feasible and accurate enough to allow for reliable calibration and prediction. MSS was initially developed in a systems biology context [29–31] and has been successfully applied to calibration and prediction of epidemics models [27,32]. This subsection will briefly summarize MSS; for full details, we refer the reader to the original publications [28–31].

Given accumulated observations *y*_1_, …, *y*_*i*_, the probability distribution for the epidemic states is called the ‘belief state’ and we denote it with *Π*(·|*y*_1_, *y*_2_, …, *y*_*i*_) at time *t*_*i*_. The belief state *Π* is a probability distribution assigning each state *ν*_*i*_ its probability conditioned on previous observations, *Π*(*ν*_*i*_ | *y*_1_, *y*_2_, …, *y*_*i*_). By conditioning on the epidemic state at time *t*_*i*−1_, denoted by *ν*_*i*−1_, and the epidemic state *ν*_*i*_ at time *t*_*i*_, the probability function 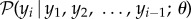 in equation (2.5) can be calculated as:2.7
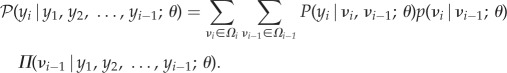
where *Ω*_*i*_ is the support of the belief state at time *t*_*i*_, and *p* is the transition probability to move from state *ν*_*i*−1_ at time *t*_*i*−1_ to state *ν*_*i*_ at time *t*_*i*_. In case of the above mentioned SIRS model, an epidemic state *ν*_*i*_ is a vector corresponding to the number of people in the compartments (*S*(*t*_*i*_), *I*(*t*_*i*_), *R*(*t*_*i*_)). *P* is the observation probability mapping the state *ν*_*i*_ to the observation *y*_*i*_ incorporating any additional uncertainty in the data collection such as reporting errors. The observation probability *P* for the new cases *y*_*i*_ is assumed to be normally distributed with a mean *ν*^(I)^_*i*_ − *ν*^(I)^_*i*−1_ + R*_i_ and variance 10 (as previously assumed in MSSa version in Zimmer *et al.* [27]).

As in previous work [30–32], we employ a linear noise approximation (LNA) method to approximate the transition probability *p* (of equation (2.6)). The LNA assumes that the probability distribution of *ν*_*i*_ | *ν*_*i*−1_ can be properly approximated by a normal distribution 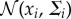 where *x*_*i*_ is the solution of the ordinary differential equation (ODE) representation of the system on the interval [*t*_*i*−1_, *t*_*i*_]2.8
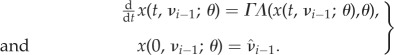
where *Γ* is a matrix describing the instantaneous change of each transition on each compartment and the vector *Λ* the rate of the instantaneous change of each transition. The initialization of the equation with 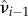 will be discussed below in equation (2.10).

The rate vector *Λ* in equation (2.7) and the instantaneous change matrix *Γ* are defined as2.9
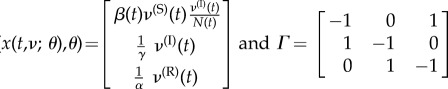
where the columns in *Γ* correspond to the transitions (namely becoming infected, recovering and loosing immunity) and the rows in *Γ* correspond to the compartments (namely *S*, *I* and *R*). The state *ν*_*i*_ consists of the number of susceptibles *ν*^(S)^_*i*_, infected *ν*^(I)^_*i*_ and recovered *ν*^(R)^_*i*_. The entry −1 in the first row and first column, hence, describes the effect of the first transition on the first compartment. Here, the first transition means becoming infected and this reduces the number of people in the first compartment by one.

According to [33,34], the covariance matrix *Σ* can be calculated by solving the following ODE system2.10
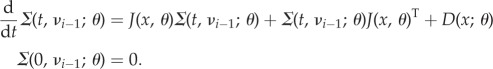
Here, *J*(*x*, *θ*) = *Γ* d/d*x* *Λ*(*x*, *θ*). *D* is matrix with the (*i*, *j*) entry equal to 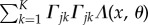.

We calculate the initial values for equation (2.7) recursively. At time *t*_*i*_, we use the previous state estimate 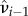 to calculate the probability to observe the current observation *y*_*i*_ as 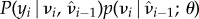. At time *t*_*i*_, we then choose the state *ν*_*i*_ as our state estimate 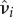 which maximizes this probability, namely2.11
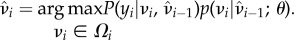
We can use this state estimate to strongly reduce the computational complexity of the second summation in equation (2.6) by using a belief state *Π*(*ν*_*i*_ | *y*_1_, …, *y*_*i*_) that yields 1 at 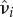 and 0 elsewhere (point distribution):
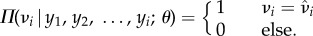
As we use parameter samples to update the prior distribution recursively (equation (2.5)), using 1000 samples of parameter vectors to forward the recursion (equation (2.5)). We additionally use a mechanism against filter degeneracy (electronic supplementary material, Mechanism against filter degeneracy).

#### 2.3.2. Prediction

The calibration is carried out iteratively according to equation (2.5) as previously described [27]. Next, we make predictions for targets denoted by *Z* based on the posterior distribution of the parameters *π* and state estimates 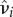 using the simulation model.2.12
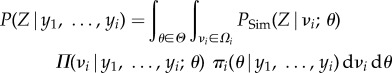
where *Θ* is the parameter vector space and *Ω*_*i*_ the state vector space.

Specifically, we sample *M*′ = 100 (as in [27]) parameter vectors *θ*^(1)^, …, *θ*^(*M*′)^ from the parameter posterior *π*_*i*_ and state vectors 
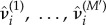
 from the belief state. For each of these *M*′ epidemic scenarios, we carry out simulations with a stochastic model *P*_Sim_ resulting in *M*′ target values *Z*^(1)^, …, *Z*^(*M*′)^. These target values are used for the posterior distributions *P* of our prediction targets *Z*.

The CDC ILI data are published after a one-week lag [15] while the Wikipedia data are available immediately. Therefore, current Wikipedia data can be used to *nowcast* the current CDC ILI data. *Nowcast* means to estimate a CDC ILI value (based on another data source such as Wikipedia data) before the CDC publishes the official value. We described an approach to estimate CDC ILI based on Wikipedia data in the ‘Wikipedia Data’ section and use 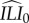 or 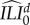 from equation (2.1) or equation (2.2) as *nowcasts*. The uncertainty around this estimate can be characterized using knowledge of its performance from past seasons (electronic supplementary material, Nowcasting).

**Figure 2.**
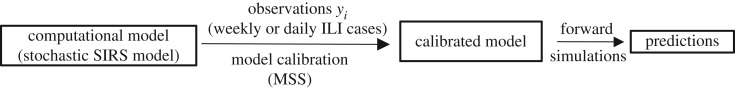
Workflow steps.

Figure 2 summarizes the main steps of our workflow.

## 3. Results

### 3.1. Evaluation scheme

We assume that CDC ILI data serves as a gold standard for influenza activity and we evaluate all predictions relative to the CDC ILI data. For all of our comparisons, we assume a one-week lag in publication of CDC ILI data. While this one-week lag in reporting of CDC data accurately reflects the reporting delay, we note that the CDC ILI data are also sometimes revised at a later date [14]. Thus, while the corrected CDC ILI data may be delayed more than a week, we conservatively assume that the final ILI data are available after 7 days.

We perform influenza forecasting retrospectively for the seasons between 2008/2009 and 2015/2016, excluding the pandemic season 2009/2010. We define an influenza season to begin at epidemic week 40 and last for 33 weeks. We assess the performance of the forecasts by calculating a log-score measure [35] which has been used for the judging of the annual CDC Influenza Prediction Challenge [4]. The log-score measure categorizes the per cent ILI in bins of size 0.1 (e.g. [1.0% ILI, 1.1% ILI], [1.1% ILI, 1.2% ILI]). The score is obtained by summing over the forecasting distribution that falls within the bin containing the true value plus the five preceding and five subsequent bins (electronic supplementary materials, Scoring System and figure A1 for details). While the log-score is a valuable instrument for comparing predictions, it is not an intuitive measure, so we also report the reduction in inter-quantile distance which we calculate as the difference between the 95%-quantile and the 5%-quantile of the posterior distribution.

We consider the predictive value of Wikipedia data in three sequential scenarios:

(a) weekly aggregated Wikipedia (ignoring the earlier availability of this data compared with CDC ILI data),
(b) weekly aggregated Wikipedia, now including its immediate availability,
(c) daily Wikipedia data, including its immediate availability.

This approach allows us to determine (a) whether our prediction suffers if we use Wikipedia as a proxy of influenza activity (and fail to take advantage of the earlier availability of Wikipedia data compared with CDC ILI data); (b) how much we gain by leveraging the earlier availability of the Wikipedia data; and (c) how much additional gain we achieve by including finer temporal resolution of Wikipedia data.

Similar to the CDC Influenza Prediction Challenge [4], we use one- to four-week predictions as our targets for our comparison. As we only use data available at the time of forecast, the forecasts early in the season are based on very few data points and the forecasts later in the season are based on the data of up to one influenza season.

Electronic supplementary material, figure A3 allows us to visualize two-week predictions of ILI and associated prediction intervals when using daily Wikipedia data.

### 3.2. Quantifying improvements in forecasting performance

**Figure 3.**
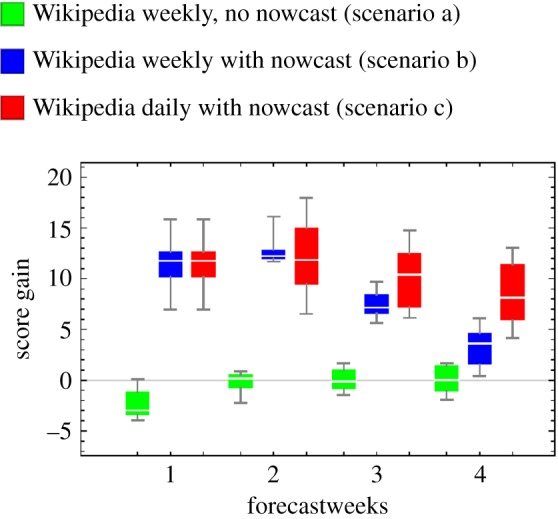
Using Wikipedia data leads to a gain in prediction of influenza. This figure summarizes influenza predictions over different years depicted in electronic supplementary material, figure A4. The following relations are significant (*p*-value < 0.05) based on the Wilcoxon signed-rank test: Weekly Wikipedia without *nowcasting* (green) is worse than the CDC ILI baseline for one-week forecasts. Weekly (blue) and daily (red) Wikipedia with *nowcasts* is always better than the CDC ILI baseline and the weekly Wikipedia without *nowcasting* (green). Daily Wikipedia (red) is better than weekly Wikipedia (blue) for three- and four-week forecasts. (Online version in colour.)

First, we examine our predictions which use weekly aggregated Wikipedia data without its *nowcasting* feature (scenario a); this is shown in green in figure 3 and electronic supplementary material, figure A4. The quality of these predictions is similar to the CDC, the ILI baseline, which demonstrates that the weekly aggregated Wikipedia data appears sufficient to replicate predictions based on CDC ILI data, but failing to leverage its early availability also eliminates any advantage of this data source.

Second, there is a large improvement when we take advantage of the more rapid availability of weekly Wikipedia data compared to the CDC ILI data (scenario b); this is shown in blue in figure 3 and electronic supplementary material, figure A4. The log-score is substantially improved compared to predictions that use only CDC ILI in all seasons.

Finally, we find that leveraging the daily Wikipedia data (scenario c) leads to additional predictive ability in most seasons, with an average gain over weekly aggregated Wikipedia data (scenario b) of 21% across the seasons we modelled; this is shown in red in figure 3 and electronic supplementary material, figure A4.

We also note that the improvement associated with use of either the weekly aggregated or daily versions of Wikipedia data is most apparent for one- and two-week predictions and is more modest for the four-week predictions. The earlier data availability transforms one-week predictions to *nowcasts* and also trims a week off of two-, three- and four-week predictions. The benefit decreases over time because the relative shortening of the prediction period decreases with increasing absolute length. Therefore, the gain associated with the earlier availability of Wikipedia data decreases as well. However, we note that even for the four-week predictions, the gain in performance associated with earlier availability of the Wikipedia data may still be important.

A closer analysis of the incremental benefit of using daily data reveals that the performance of weekly Wikipedia data (scenario b) and daily Wikipedia data (scenario c) is identical for one-week predictions. This occurs because one-week ‘predictions’ are no longer real predictions due the earlier data availability; these are *nowcasts* and the same *nowcasting* scheme is used for both (scenario b and scenario c).

Comparing using weekly Wikipedia data with using daily Wikipedia data for predictions, we note that the use of daily data is advantageous for all years and the incremental benefit of the daily data is strongest for four-week predictions with an average 409% improvement compared to weekly data. The gain in performance for three-week predictions is on average 38% with daily data performing better than weekly data in five out of seven seasons. For two-week predictions, using daily data was better than using weekly in only three out of seven seasons and performed on average 6% worse.

**Figure 4.**
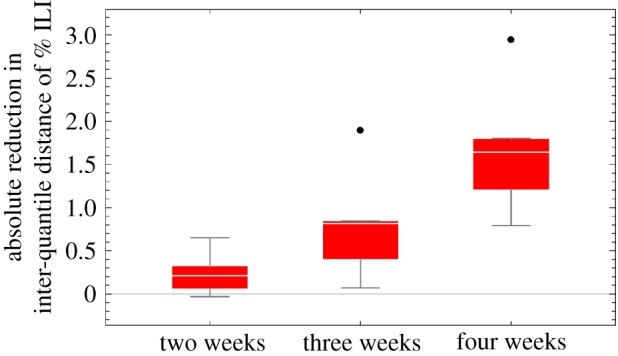
Using daily Wikipedia data versus weekly Wikipedia data reduces the inter-quantile distance of % ILI forecasts. For all seven seasons, using daily versus weekly Wikipedia data reduces the inter-quantile distance for two-, three- and four-week forecasts. The reduction using daily Wikipedia data was significant (*p*-value <0.05) for two-, three- and four-week forecasting targets based on the Wilcoxon signed-rank test. (Online version in colour.)

While the use of the log-score is a well-established approach to compare the performance of prediction systems, it is a non-intuitive measure. Therefore, we also visualize the reduction in the prediction uncertainty when using daily Wikipedia data by reporting the inter-quantile distance (between 5%- and 95%-quantile) of the posterior distribution to provide an illustration of the distribution width (electronic supplementary material, Technical details). By using daily Wikipedia data, we are able to achieve an absolute reduction in inter-quantile distance by an average 0.24% ILI for the two-week predictions, a 0.76% ILI reduction for the three-week predictions and a 1.64% ILI reduction for the four-week predictions (figure 4). This narrower posterior translates to a reduction in prediction uncertainty. We note that this improvement does not lead to more frequent prediction failure (i.e. true value outside of confidence interval) since for both weekly and daily data, the coverage for all targets remains above 90% (electronic supplementary material, table A1).

**Figure 5.**
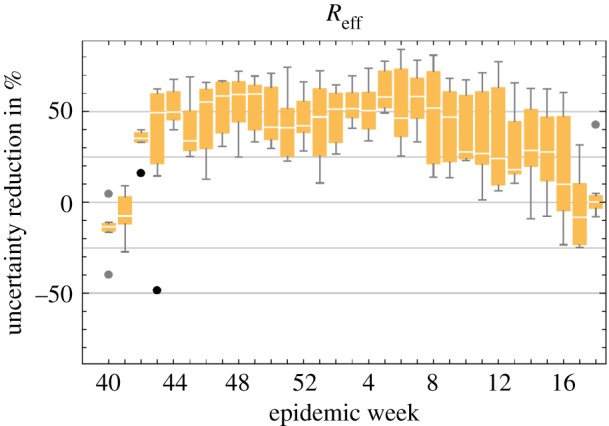
Using daily instead of weekly Wikipedia data reduces parameter uncertainty. Relative reduction in inter-quantile distance (5%- to 95%-quantile) for estimates of the effective reproductive number, *R*_eff_, over the seven seasons indicates using daily versus weekly Wikipedia data reduces parameter uncertainty.

As noted previously, the use of daily data (compared with weekly aggregated data) produces the greatest gains for the three- and four-prediction horizon (figures 3 and electronic supplementary material, figure A4, the comparison between the red and blue bars). To further investigate the source of this benefit, we analysed the posterior distributions of the parameters. Since the true values of parameters are not known, we cannot use a log-score measure, and instead we use inter-quantile distance to evaluate the parameter distributions. There is a substantial reduction in parameter uncertainty for *R*_eff_ (figure 5) which can be largely attributed to an uncertainty reduction in the time-varying *R*_0_(*t*) (electronic supplementary material, figure A5). This reduction in parameter uncertainty in *R*_eff_ likely explains the reduction in prediction uncertainty when using daily Wikipedia data.

All of our model-based predictions can be found in the electronic supplementary material, Prediction results in influenza prediction challenge format; we use the same format as the CDC Influenza Prediction Challenge to facilitate replication and comparison by other modellers [4].

## 4. Discussion

In this study, we demonstrate and quantify the improvement in model-based influenza prediction that may be achieved by using an immediately available data source rather than the CDC ILI surveillance system which reports weekly aggregated data after a one-week delay (figure 3 and electronic supplementary material, figure A4). Further, we find that the temporal resolution of observations matters for prediction quality, and an additional gain in precision can be achieved by using daily-resolved data (versus weekly aggregated data) (figure 4). As all our forecasts are available in the electronic supplementary material, we anticipate that this work will be compared to future approaches for model-based influenza prediction (electronic supplementary material, Prediction results in influenza prediction challenge format).

Much recent research activity has focused on short- and medium-term predictions of influenza activity [2,36] and the importance of *nowcasting* [6–15,37]. While several previous studies have used similar indirect sources of influenza activity data for forecasting [16,19,24], none of these studies have used daily data or quantified the improvements associated with using these more readily available data sources. Other work [21,22,38,39] has used data with a daily resolution as a basis for forecasting, but these studies have not evaluated the performance over several seasons and do not report gains in prediction performance associated with the higher resolution observation frequency.

Our study clearly shows that influenza prediction can be improved by using a data source that is updated daily and available in near real-time. On average, over the seven seasons we studied, the improvements of daily versus weekly Wikipedia data were up to 409% for four-week predictions (measured in log-score) and 38% for three-week predictions. We note that Wikipedia data are readily available at a daily resolution, so this gain in prediction performance can be achieved without any further cost in data collection.

As our study uses Wikipedia data, it suffers from similar limitations to other modelling studies that use Internet search data to predict influenza activity. For example, it is possible that varying intensity of search activity over a season may compromise the utility of this data source [40]. A Wikipedia-specific limitation is that global article searches for English language articles are aggregated, and searches originating specifically in the USA are not available. This aggregation could erode the value of this data source for predicting specifically US epidemics.

We also note that the improvement associated with using daily-resolved data has been achieved using our calibration and prediction framework as in Zimmer *et al.* [27] and in the previously described methods. We did not investigate whether similar improvements would be seen if other calibration and prediction approaches were used.

We found that the strongest gain in performance associated with use of daily data compared to weekly data is for the three- and four-week forecasting horizons (figures 3 and 4). This suggests that daily-resolved data may help with resource planning given that this horizon seems feasible for public health planning. Whether such improvements in prediction allow for the deployment of more efficient or effective interventions is not directly addressed by our current investigation.

In summary, we find that the use of near real-time daily Internet search data improves the precision of short- and medium-term forecasts of influenza activity. Given the free and ubiquitous nature of this type of information, we expect that future predictions which leverage data at finer temporal resolution and with limited reporting delay will produce epidemic predictions with less uncertainty to better inform reactive public health policy.

## Supplementary Material

Supporting Information

## Supplementary Material

Prediction results in FluChallenge format

## Supplementary Material

Computer code and results

## Data accessibility

All data is accessible as part of the electronic supplementary material.
