# Supplementary material for: Use of daily Internet search query data improves real-time projections of influenza epidemics
Source: J R Soc Interface. 2018 Oct 10;15(147):20180220. doi: 10.1098/rsif.2018.0220 (PMC6228485; doi:10.1098/rsif.2018.0220)
Supplement: Supporting Information [file rsif20180220supp1.pdf]

Use of daily internet search query data improves real-time projections of influenza epidemics

Christoph Zimmer*[1,3], Sequoia I. Leuba[1], Reza Yaesoubi[2], and Ted Cohen[1]

[1] Epidemiology of Microbial Diseases, Yale School of Public Health, New Haven, CT, USA
[2] Health Policy and Management, Yale School of Public Health, New Haven, CT, USA
[3] Bosch Center for Artificial Intelligence, Robert Bosch GmbH, Renningen, Germany
Email: christoph.zimmer@yale.edu; christoph.zimmer@de.bosch.com
*Corresponding author

Journal of the Royal Society Interface

Supporting Information (SI)

Humidity data

In Zimmer et al. [26], we independently reconstructed the specific humidity dataset following the method detailed by the Text S1 of Yang et al. [19]. The specific humidity dataset was developed from the primary forcing dataset from Phase 2 of the North American Land Data Assimilation System (NLDAS-2) [40]. This data was derived through the National Center for Environmental Prediction North American Regional Reanalysis. The hourly data are available on a 0.125° grid from 1979 to present. We extracted the specific humidity data for 121 cities and then averaged the hourly data to developed a daily climatology for each city from 1979 to 2016.

Averaging the yearly data over the years 1979 until 2016 gives an average yearly humidity profile for each city, and we used the average over all the cities as a yearly national humidity profile.

Nowcasting

Here, we describe how to characterize the uncertainty around our *nowcasts*. As Wikipedia data is available one week before the CDC publishes their ILI data, the Wikipedia data can be used to *nowcast* this week's CDC ILI (which will be published following week).

We described how to calculate a Wikipedia proxy of CDC ILI in the Wikipedia data section in the main text; here, we describe how we calculate a posterior distribution for this estimate which can then be used to quantify the uncertainty around the estimate that is used for *nowcasting*.

We denote the epidemic week of data collection with j and calculate the residuals between the Wikipedia-based estimate for CDC's ILI, $\widehat{ILI}_0^{(j)}$ (Equation (1)), and the true CDC ILI value published a week later, $ILI_0^{(j)}$ as

$$\rho_j = \widehat{ILI}_0^{(j)} - ILI_0^{(j)}$$

and use a density estimation to describe the probability of the CDC ILI given the weekly Wikipedia-based ILI projection, $\widehat{ILI}_0$, as

$$f(ILI_0|\widehat{ILI}_0) = f(\widehat{ILI}_0 + \rho|\widehat{ILI}_0) = \frac{1}{n\ h} \sum_{j \in J} k\left(\frac{\rho - \rho_j}{h}\right) \tag{13}$$

with a bandwidth h, a smoothing kernel k, and a set of epidemic weeks from past years J. The bandwidth h in Equation (13) is automatically chosen by Mathematica [41], and the kernel is a Gaussian kernel. In the case of daily Wikipedia data, the density is centered around the sum over the seven daily projections. If not all of these seven daily projections are available (e.g. if the recording just started at the beginning of the season), we multiply the last daily projection of the week by seven.

The range of j extends from 2008 until the year under consideration and begins for each year in epidemic week 40 and continues for 34 epidemic weeks. Note that we need 34 weeks here (even though we consider only 33 weeks in the main text for the evaluation) as *nowcasting* is always performed for the next week.

We note that previous observations are not available for the first epidemic season, so we chose to use the same *nowcasting* posterior for 2008 as for 2010. Therefore, our *nowcasting* targets of the 2008 year contain information that was not previously available. We note that this is not an issue for using our described approach in the future as data from past seasons are available now.

Mechanism against filter degeneracy

We use $M = 1000$ parameter vector samples whenever we perform the update in Equation (6). After a few of these update steps, it occurs that the weight of all except very few (potentially only one) parameter vectors are orders of magnitudes lower than the highest. This effectively means that the parameter posterior is degenerated to a point distribution – a common phenomenon referred to as filter degeneracy [42,43]. We use the same mechanism as in [26] to overcome this problem. We first assign a weight $w_m = \pi_i(\theta_m)$ for each parameter vector. Next, we calculate a measure of (non-) degeneracy called effective sample size [43]

$$\hat{N}_{\text{eff}} = \frac{1}{\sum_{m=1}^{M} \tilde{w}_m^2}. \tag{14}$$

where $\tilde{w}$ the normalized weights. Equal weights lead to $\hat{N}_{\text{eff}} = M$, and the other extreme of all weight being on one parameter vector leads to $\hat{N}_{\text{eff}} = 1$. As in [26], we chose a threshold of $N_C = M/2$ to detect filter degeneracy by $\hat{N}_{\text{eff}} \leq N_C$.
If filter degeneracy is detected, we construct a parameter distribution density estimate as

$$\hat{f}(\theta) = \frac{1}{\tilde{M}} \det(H)^{-\frac{1}{2}} \sum_{l=1}^{\tilde{M}} K\left(\det(H)^{-\frac{1}{2}}(\theta - \theta_l^{'})\right), \tag{15}$$

where $\theta_1^{'}, \ldots, \theta_{\tilde{M}}^{'}$ are re-sampled with replacement from $\theta_1, \ldots, \theta_M$ weighted by $w_1, \ldots, w_M$. The bandwidth matrix H controls the smoothness of the density estimate and K is the multivariate kernel (e.g. Gaussian).
Lastly, we sample M new parameter vectors $\theta_1^{'}, \ldots, \theta_M^{'}$ from $\hat{f}$ and calculate their new weights $\bar{w}_m$ as

$$\bar{w}_m = \prod_{k=1}^{i} \mathcal{P}(y_k|y_1, \ldots, y_{k-1}, \theta_m^{'}). \tag{16}$$

Technical Details

- We calculate inter-quantile distances as the distance between the 5%-quantile and 95%-quantile of the posterior distribution. When available (for forecasts), we use the forecasting data structure of the CDC's Influenza Prediction Challenge which can be found in the SI file "Prediction results in

Influenza Prediction Challenge format". As this data structure is based on bins with a highest bin ranging from 13% ILI to 100% ILI, we choose 15% ILI as the 95% quantile if the 95% quantile fall into this bin.

- One could think of different ways for the daily Wikipedia data aggregation such as

$$\overline{ILI}_0^{d,*} = b_0/7 + b_1/7 \cdot x_1^d + b_2/7 \cdot x_2^d + b_3/7 \cdot x_3^d + b_4/7 \cdot x_4^d + b_5/7 \cdot x_5^d + b_6/7 \cdot \widetilde{ILI}_-$$

  However, Figure A6 indicates that this does not lead to a significant gain.

Scoring system

Following the scoring standards for the CDC Influenza Prediction Challenge [4], we use a logarithmic score to measure prediction performance [34]. The adoption of this scoring system is based on our desire to facilitate the ability of other modelers to compare predictions. The score is based on the following approach: first, bins of width 0.1 for the percentage of all patient visits related to ILI are created, second, we calculate the probability that the posterior falls into the bin containing the true value and five preceeding (lower) and five subsequent (higher) bins (Figure A1). Next, the natural logarithm of this probability count is calculated. The log-score is defined as this probability count if it is larger than -10, and is defined as -10 otherwise.

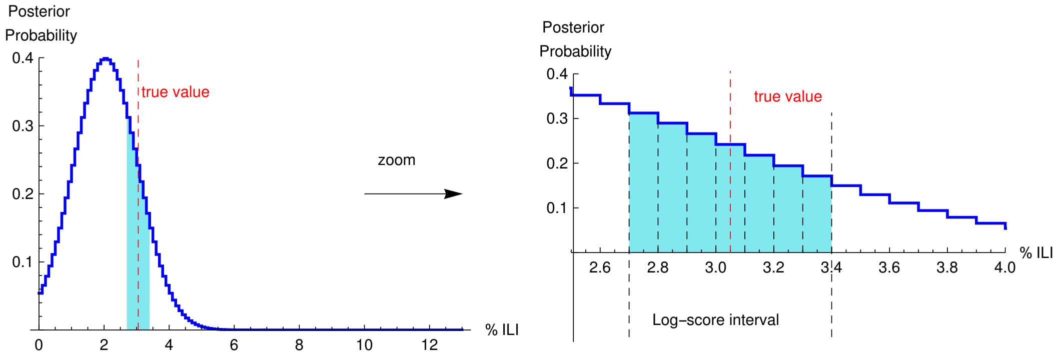

Figure A1: **Illustration of the log-score evaluation system:** The left panel is the full picture and the right panel zooms in on the prediction posterior distribution. The x-axis is the % ILI, and the y-axis is the posterior probability. The posterior distribution is binned in steps of 0.1% ILI. The log-score as used in the CDC's Influenza Prediction Challenge [4] sums the probability of the bin that contains the true value and the preceeding and the following bins. The natural logarithm of this sum is used as a score.

SI figures

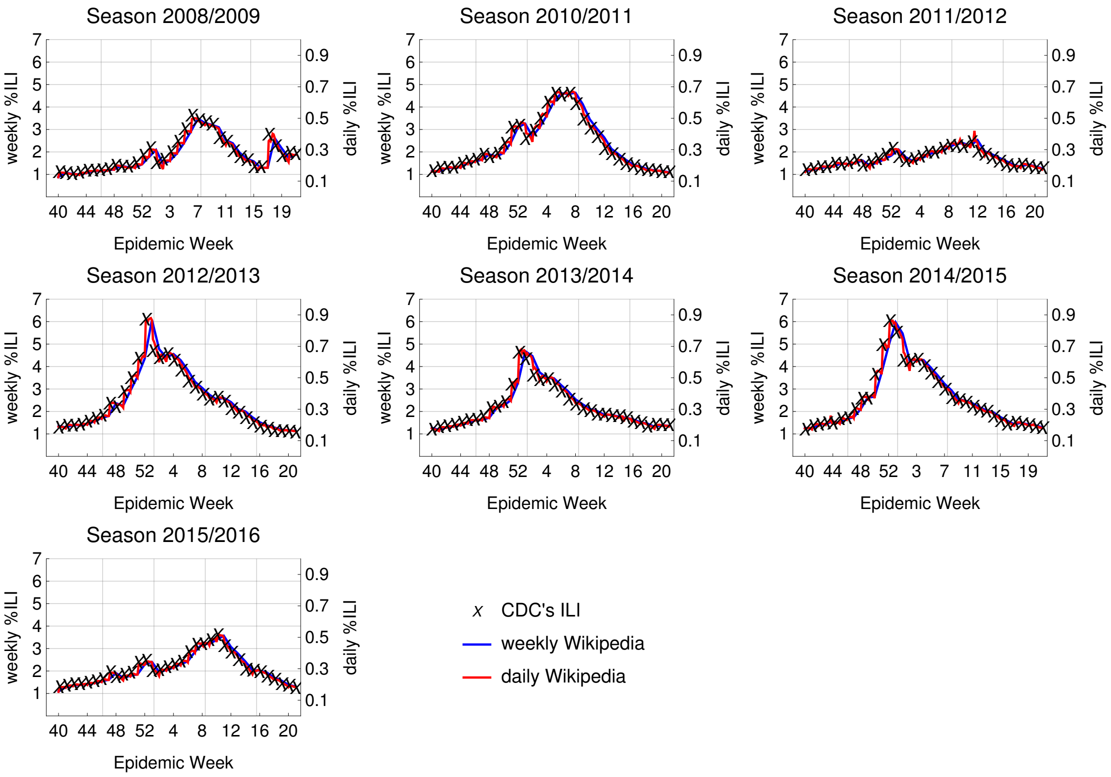

Figure A2: **Wikipedia data in daily or weekly resolution matches CDC ILI well:** Each panel shows one year, the x-axis displays the epidemic week, and the y-axis the % ILI. The black crosses are CDC ILI, the blue line is the Wikipedia proxy with weekly-aggregated data (corresponding to the left y-axis), and the red line is the Wikipedia proxy with daily data (corresponding to the right y-axis).

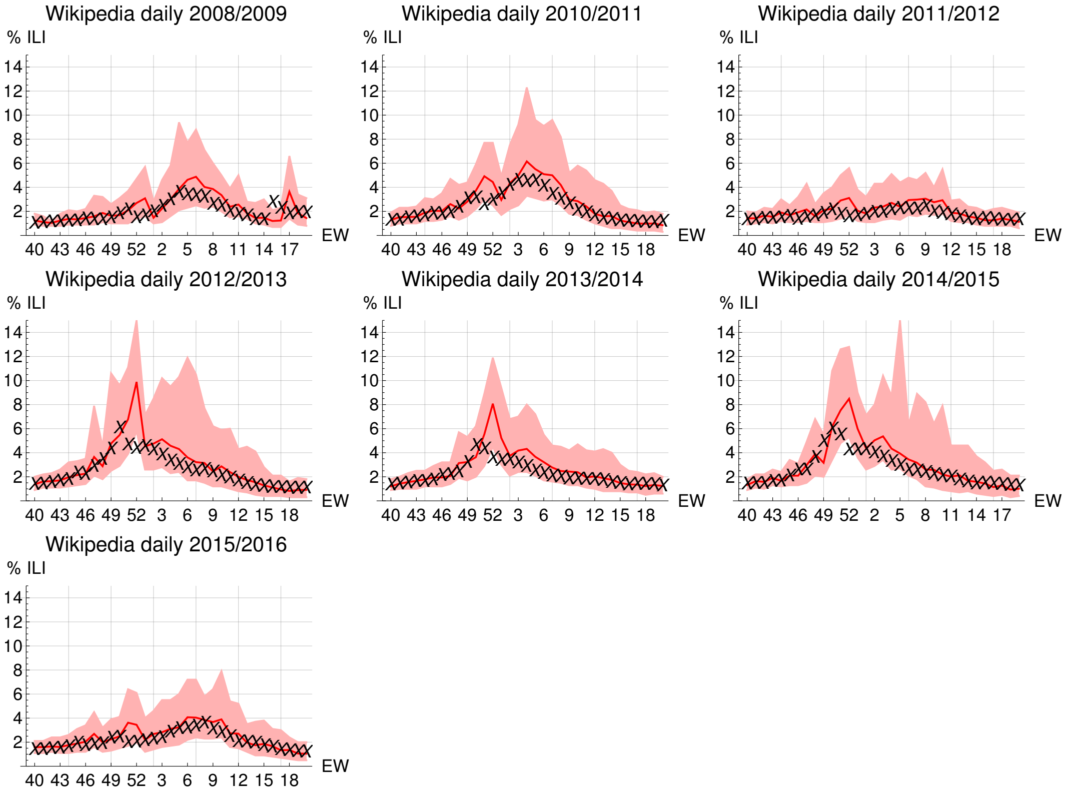

Figure A3: **Daily Wikipedia data can be used to predict CDC ILI well:** The black crosses depict the true values of percent of physician visits related to ILI observed over the course of the epidemic. The red line represents sequential point predictions and the red-shaded area represents the 90% prediction interval. The x-axis is the epidemic week (EW) and the y-axis is the % of physician visits related to ILI. This figure shows the performance of our prediction framework for 2-week predictions according to the CDC Influenza Prediction Challenge (e.g. being in epidemic week 50, CDC data is available for epidemic week 49 (due to the reporting delay) and we predict epidemic week 51). Due to the earlier availability of Wikipedia data, these predictions are essentially one-week predictions.

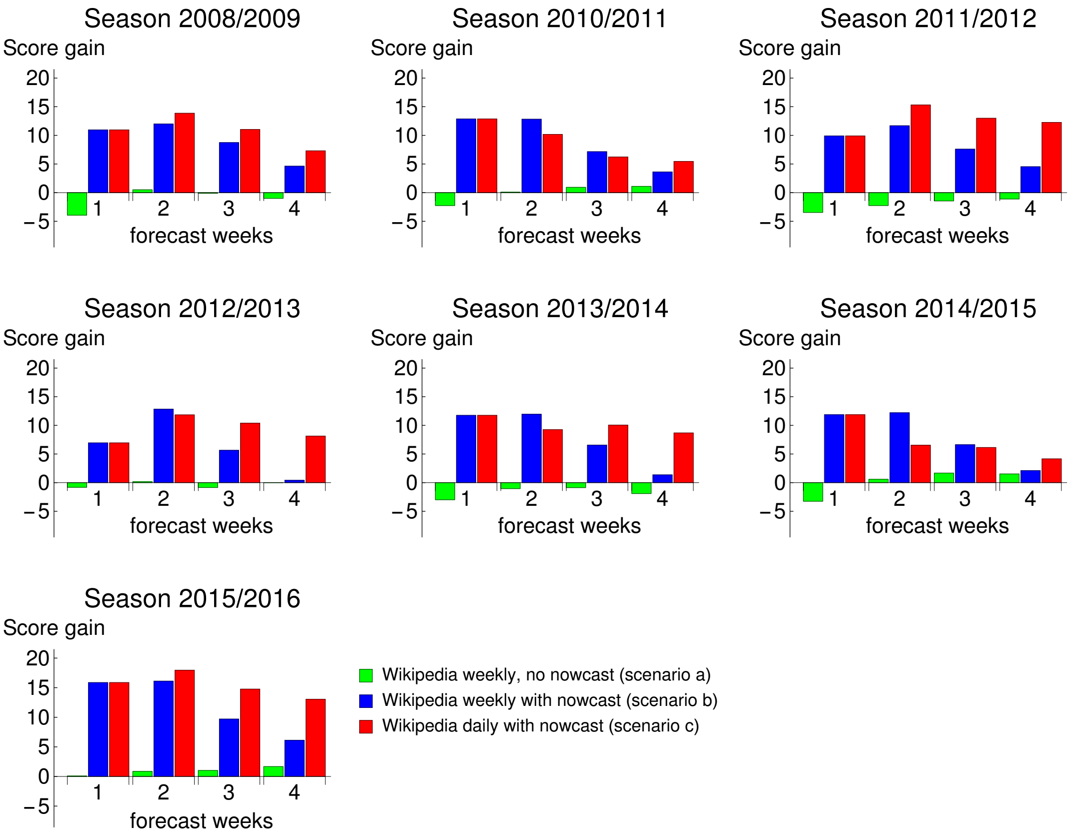

Figure A4: **Using daily Wikipedia data or weekly Wikipedia data leads to a score gain in epidemic predictions:** In each panel, the log-score associated with predictions based on Wikipedia data is compared to the log-score associated with predictions using only CDC ILI by calculating the differences between the CDC ILI log-score and the respective Wikipedia log-score for different prediction horizons.

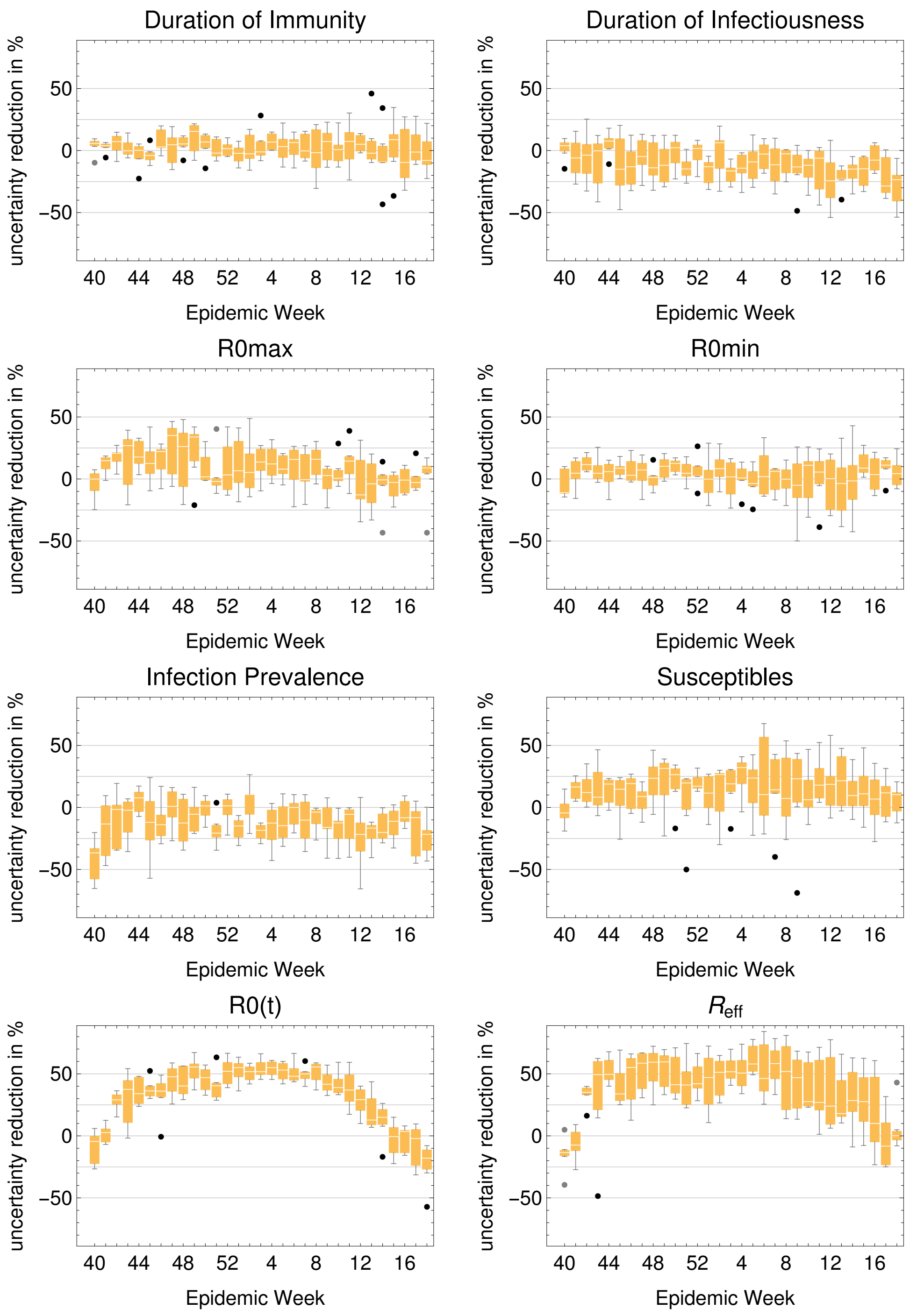

Figure A5: **Using daily instead of weekly Wikipedia data reduces parameter uncertainty:** In each panel, the x-axis displays the epidemic week and the yellow bar the relative reduction in inter-quantile distance (5%- to 95%-quantile) over the seasons 2008/09 to 2015/16 seasons (without the pandemic season of 2009/10)

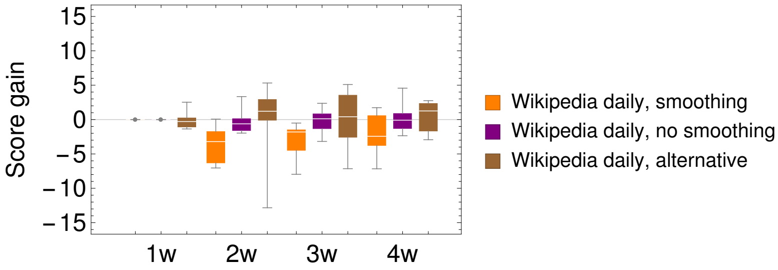

Figure A6: **Performance comparison of the different approaches on smoothing daily Wikipedia data:** We set the partial smoothing approach as used in the main text and defined in the "Daily search data" section as baseline and compare the log-score gain or loss of the other approaches to it: smoothing, no smoothing and the alternative described in SI "Technical details". We see negative score gains for most forecasting targets, which means that the partial smoothing approach used in the main text is a reasonable choice. However, the only significant (to p-value < 0.05) results, using a Wilcoxon signed-rank test, are those for smoothing for 2- and 3-week forecasts which might be due to the limited number of seasons currently available (2008/09 to 2015/16 without the pandemic season of 2009/10).

SI tables

Data Scenario	1-week	2-week	3-week	4-week
CDC ILI	97%	98%	99%	100%
weekly Wikipedia, no nowcast	97%	98%	100%	100%
weekly Wikipedia with nowcast	93%	97%	98%	100%
daily Wikipedia with nowcast	93%	94%	95%	98%

Table A1: **All targets are covered above 90% indicating that using daily Wikipedia data does not lead to more frequent prediction failure:** Coverage was defined as the percent of true values that are within the 90% posterior prediction interval for different forecast horizons.

SI files

Prediction results in Influenza Prediction Challenge format

All forecasts (1-4 weeks) for all approaches (CDC ILI as baseline, Wikipedia without *nowcast*, Wikipedia-weekly, Wikipedia-daily) for all seasons can be found in the same format as the 2016/2017 Influenza Prediction Challenge as a compressed file.

Computer code and results

All computer code necessary to reproduce our analysis can be found compressed as a compressed file. This code contains components of Wikipedia data [25] and NASA humidity data [40]. Re-use only permitted according to their policies.

References

[1] L. Simonsen, M. J. Clarke, G. D. Williamson, D. F. Stroup, N. H. Arden, and L. B. Schonberger. The impact of influenza epidemics on mortality: Introducing a severity index. *American Journal of Public Health*, 87, 1997.

[2] J. P. Chretien, D. George, J. Shaman, R. A. Chitale, and F. E. McKenzie. Influenza forecasting in human populations: a scoping review. *PLoS ONE*, 9(4):e94130, 2014.

[3] Centers for Disease Control and Prevention. Disease burden of influenza. *https://www.cdc.gov/flu/about/disease/burden.htm*, Accessed May 30th, 2017.

[4] Epidemic Prediction Initiative BETA. *https://predict.phiresearchlab.org/legacy/flu/evaluation.html*, Accessed 2017-02-02, 2016.

[5] Centers for Disease Control and Prevention. Flu activity and surveillance. *https://www.cdc.gov/flu/weekly/fluactivitysurv.htm*, Accessed May 31st, 2017.

[6] J. Ginsberg, M. H. Mohebbi, R. S. Patel, L. Brammer, M. S. Smolinski, and L. Brilliant. Detecting influenza epidemics using search engine query data. *Nature Letters*, 457, 2009.

[7] J. R. Ortiz, H. Zhou, D. K. Shay, K. M. Neuzil, A. L. Fowlkes, and C. H. Goss. Monitoring influenza activity in the united states: A comparison of traditional surveillance systems with google flu trends. *PlosONE*, 6, 2011.

[8] T. Preis and H. S. Moat. Adaptive nowcasting of influenza outbreaks using google searches. *R. Soc. open sci.*, 1, 2014.

[9] S. Cho, C. H. Sohn, M. W. Jo, S.-Y. Shin, J. H. Lee, S. M. Ryoo, W. Y. Kim, and D.-W. Seo. Correlation between national influenza surveillance data and google trends in south korea. *PlosONE*, 8, 2013.

[10] M. Kang, H. Zhong, J. He, S. Rutherford, and F. Yang. Using google trends for influenza surveillance in south china. *PlosONE*, 8, 2013.

[11] L. J. Martin, B. E. Lee, and Y. Yasui. Google flu trends in canada: a comparison of digital disease surveillance data with physician consultations and respiratory virus surveillance data, 2010 – 2014. *Epidemiol. Infect.*, 144:325 – 332, 2016.

[12] D. Lazer, R. Kennedy, G. King, and A. Vespignani. The parable of google flu: Traps in big data analysis. *Science*, 343:1203 – 1205, 2014.

[13] D. A. Broniatowski, M. J. Paul, and M. Dredze. National and local influenza surveillance through twitter: An analysis of the 2012-2013 influenza epidemic. *Plos ONE*, 8, 2013.

[14] M. J. Paul, M. Dredze, and D. Broniatowski. Twitter improves influenza forecasting. *Plos Current Outbreaks*, 1, 2014.

[15] D. J. McIver and J. S. Brownstein. Wikipedia usage estimates prevalence of influenza-like illness in the united states in near real-time. *Plos Computational Biology*, 10, 2014.

[16] K. S. Hickmann, G. Fairchild, R. Priedhorsky, N. Generous, J. M. Hyman, A. Deshpande, and S. Y. Del Valle. Forecasting the 2013 – 2014 influenza season using wikipedia. *Plos Computational Biology*, 11, 2015.

[17] M. Biggerstaff, D. Alper, M. Dredze, S. Fox, I. Chun-Hai Fung, K. S. Hickmann, B. Lewis, R. Rosenfeld, J. Shaman, M.-H. Tsou, P. Velardi, A. Vespignani, and L. Finelli. Results from the centers for disease control and prevention's predict the 2013 – 2014 influenza season challenge. *BMC Infectious Diseases*, 16, 2016.

[18] J. Shaman, A. Karspeck, W. Yang, J. Tamerius, and M. Lipsitch. Real-time influenza forecasts during the 2012-2013 season. *Nat Commun*, 4:2837, 2013.

[19] W. Yang, A. Karspeck, and J. Shaman. Comparison of filtering methods for the modeling and retrospective forecasting of influenza epidemics. *PLOS Computational Biology*, 10:e1003583, 2014.

[20] W. Yang, B. J. Cowling, E. H. Lau, and J. Shaman. Forecasting Influenza Epidemics in Hong Kong. *PLoS Comput. Biol.*, 11(7):e1004383, Jul 2015.

[21] J. B. Ong, M. I. Chen, A. R. Cook, H. C. Lee, V. J. Lee, R. T. Lin, P. A. Tambyah, and L. G. Goh. Real-time epidemic monitoring and forecasting of H1N1-2009 using influenza-like illness from general practice and family doctor clinics in Singapore. *PLoS ONE*, 5(4):e10036, 2010.

[22] V. DUKIC, H.F. LOPES, and N. G. POLSON. Tracking Epidemics With Google Flu Trends Data and a State-Space SEIR Model. *Journal of the American Statistical Association*, 107, 2012.

[23] C. Viboud, P. Boëlle, F. Carrat, A. Valleron, and A. Flahault. Prediction of the spread of influenza epidemics by the method of analogues. *American Journal of Epidemiology*, 158, 2003.

[24] L. C. Brooks, D. C. Farrow, S. Hyun, R. J. Tibshirani, and R. Rosenfeld. Flexible modeling of epidemics with an empirical bayes framework. *Plos Computational Biology*, 11, 2015.

[25] Page view statistics for wikimedia projects. *https://dumps.wikimedia.org/other/pagecounts-raw/*, Accessed Jan-March, 2017.

[26] C. Zimmer, S. I. Leuba, R. Yaesoubi, and T. Cohen. Accurate quantification of uncertainty in epidemic parameter estimates and predictions using stochastic compartmental models. *Statistical Methods in Medical Research*, In Press.

[27] D.T. Gillespie. A general method for numerically simulating the stochastic time evolution of coupled chemical reactions. *Journal of Computational Physics*, 22 (4):403–434, 1976.

[28] C. Zimmer and S. Sahle. Parameter estimation for stochastic models of biochemical reactions. *Journal of Computer Science & Systems Biology*, 6:011–021, 2012.

[29] C. Zimmer and S. Sahle. Deterministic inference for stochastic systems using multiple shooting and a linear noise approximation for the transition probabilities. *IET Systems Biology*, 9:181 – 192, 2015.

[30] C. Zimmer. Reconstructing the hidden states in time course data of stochastic models. *Mathematical BioSciences*, 269:117 – 129, 2015.

[31] C. Zimmer, R. Yaesoubi, and T. Cohen. A likelihood approach for real-time calibration of stochastic compartmental epidemic models. *PLOS Computational Biology*, 13, 2017.

[32] Philipp Thomas, Hannes Matuschek, and Ramon Grima. Intrinsic noise analyzer: a software package for the exploration of stochastic biochemical kinetics using the system size expansion. *PloS one*, 7(6):e38518, 2012.

[33] Nicolaas Godfried Van Kampen. *Stochastic processes in physics and chemistry*, volume 1. Elsevier, 1992.

[34] T. Gneiting and A. E. Raftery. Strictly proper scoring rules, prediction, and estimation. *Journal of the American Statistical Association*, 102:359 – 378, 2007.

[35] E. O. Nsoesie, J. S. Brownstein, N. Ramakrishnan, and M. V. Marathe. A systematic review of studies on forecasting the dynamics of influenza outbreaks. *Influenza and Other Respiratory Viruses*, 2013.

[36] B. Nunes, I. Natàriob, and M. L. Carvalho. Nowcasting influenza epidemics using non-homogeneous hidden markov models. *Statistics in Medicine*, 32, 2013.

[37] X. Jiang, G. Wallstrom, G. F. Cooper, and M. M. Wagner. Bayesian prediction of an epidemic curve. *Journal of Biomedical Informatics*, 42:90 – 99, 2009.

[38] C.J. Rhodes and T.D. Hollingsworth. Variational data assimilation with epidemic models. *Journal of Theoretical Biology*, 258:591 – 602, 2009.

[39] D. R. Olson, K. J. Konty, M. Paladini, C. Viboud, and L. Simonsen. Reassessing google flu trends data for detection of seasonal and pandemic influenza: A comparative epidemiological study at three geographic scales. *Plos Computational Biology*, 9, 2013.

[40] Y Xia and et al. Nldas primary forcing data l4 hourly 0.125 x 0.125 degree v002. *, Greenbelt, Maryland, USA, Goddard Earth Sciences Data and Information Services Center (GES DISC)*, Accessed August 8th 2016.

[41] Mathematica, version 10.4. *Wolfram Research, Inc.*, Champaign, IL, 2015.

[42] A. E. Raftery and L. Bao. Estimating and projecting trends in hiv/aids generalized epidemics using incremental mixture importance sampling. *Biometrics*, 66:1162–1173, 2010.

[43] M. S. Arulampalam, S. Maskell, N. Gordon, and T. Clapp. A tutorial on particle filters for online nonlinear/non-gaussian bayesian tracking. *IEEE TRANSACTIONS ON SIGNAL PROCESSING*, 50:174–188, 2002.
