# Supplementary material for: Use of daily Internet search query data improves real-time projections of influenza epidemics
Source: J R Soc Interface. 2018 Oct 10;15(147):20180220. doi: 10.1098/rsif.2018.0220 (PMC6228485; doi:10.1098/rsif.2018.0220)
Supplement: Computer code and results [file rsif20180220supp3.gz › Code-and-results/plots/bc3.pdf]

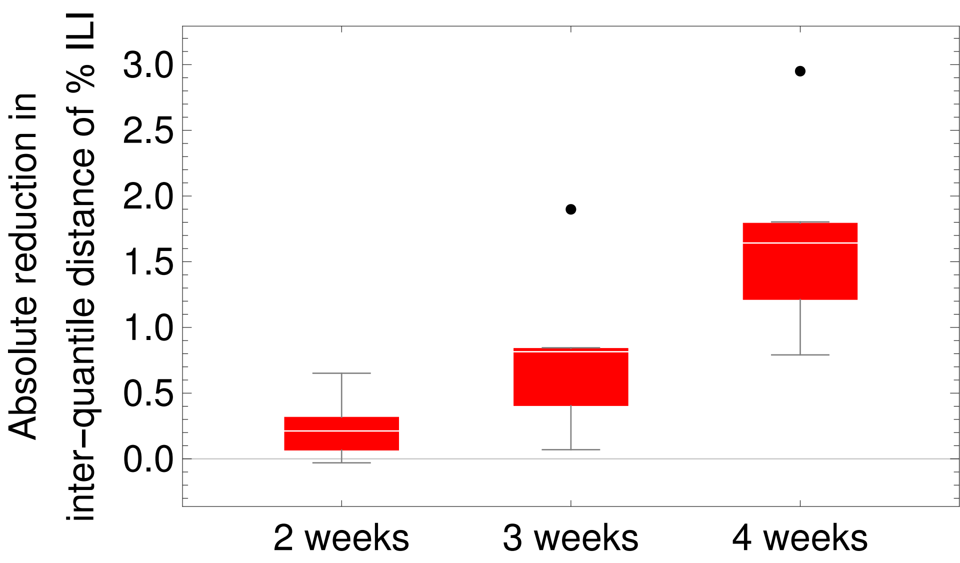
Absolute reduction in
inter–quantile distance of % ILI
3.0
2.5
2.0
1.5
1.0
0.5
0.0
2 weeks
3 weeks
4 weeks
