# Supplementary material for: Use of daily Internet search query data improves real-time projections of influenza epidemics
Source: J R Soc Interface. 2018 Oct 10;15(147):20180220. doi: 10.1098/rsif.2018.0220 (PMC6228485; doi:10.1098/rsif.2018.0220)
Supplement: Computer code and results [file rsif20180220supp3.gz › Code-and-results/plots/log-score-illust.pdf]

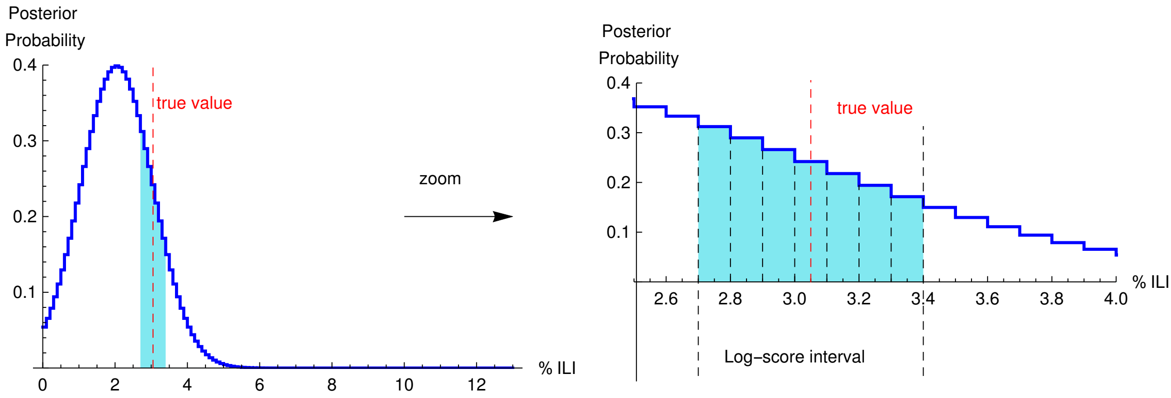

Posterior
Probability
0.4
0.3
0.2
0.1
true value
0
2
4
6
8
10
12
% ILI
zoom
Posterior
Probability
0.4
0.3
0.2
0.1
true value
2.6
2.8
3.0
3.2
3.4
3.6
3.8
4.0
% ILI
Log−score interval
