# Supplementary material for: Use of daily Internet search query data improves real-time projections of influenza epidemics
Source: J R Soc Interface. 2018 Oct 10;15(147):20180220. doi: 10.1098/rsif.2018.0220 (PMC6228485; doi:10.1098/rsif.2018.0220)
Supplement: Computer code and results [file rsif20180220supp3.gz › Code-and-results/plots/allyears.pdf]

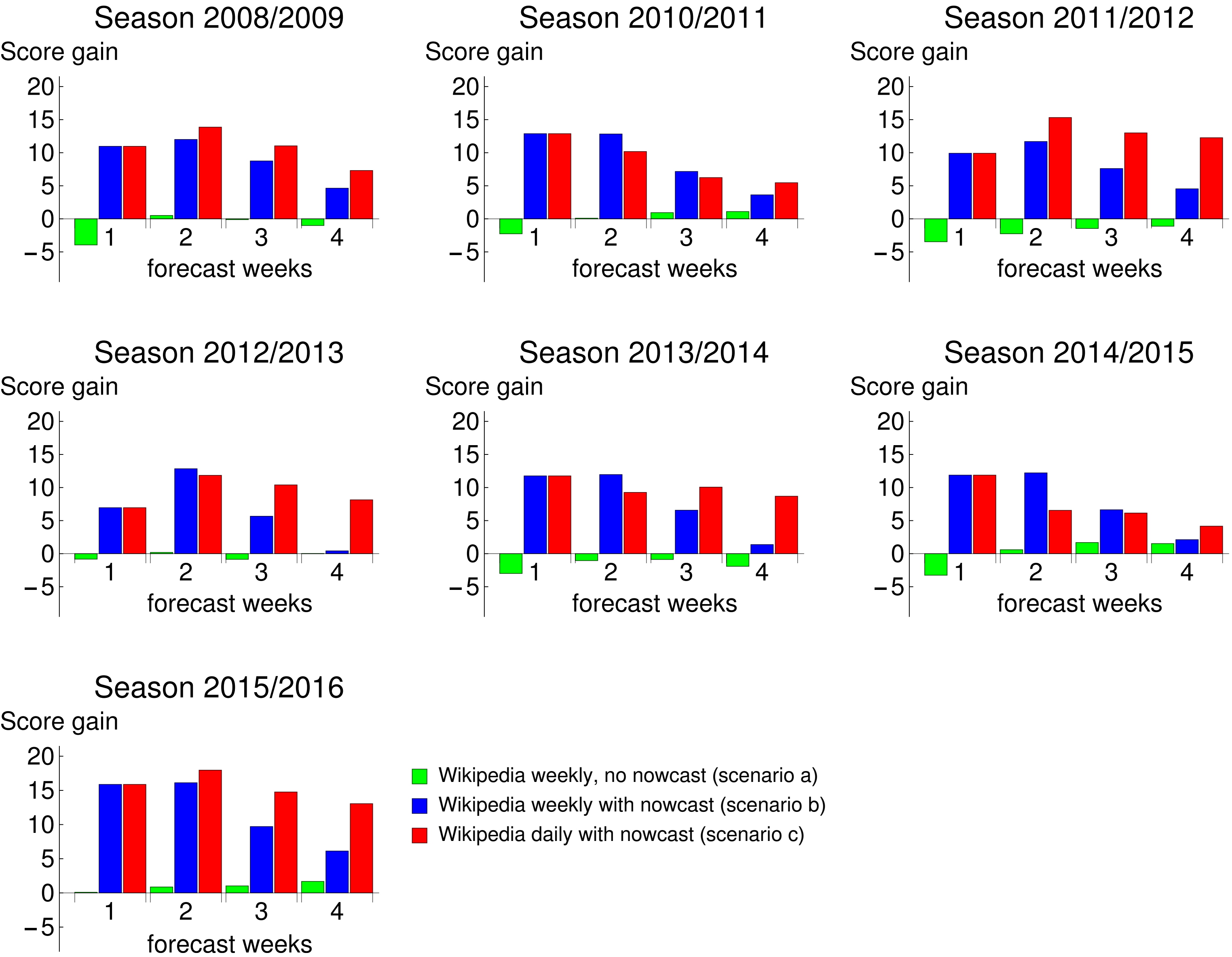

Season 2008/2009
Season 2010/2011
Season 2011/2012
Season 2012/2013
Season 2013/2014
Season 2014/2015
Season 2015/2016
Score gain
forecast weeks
20
15
10
5
0
−5
1
2
3
4
Wikipedia weekly, no nowcast (scenario a)
Wikipedia weekly with nowcast (scenario b)
Wikipedia daily with nowcast (scenario c)
