# Supplementary material for: Use of daily Internet search query data improves real-time projections of influenza epidemics
Source: J R Soc Interface. 2018 Oct 10;15(147):20180220. doi: 10.1098/rsif.2018.0220 (PMC6228485; doi:10.1098/rsif.2018.0220)
Supplement: Computer code and results [file rsif20180220supp3.gz › Code-and-results/plots/params.pdf]

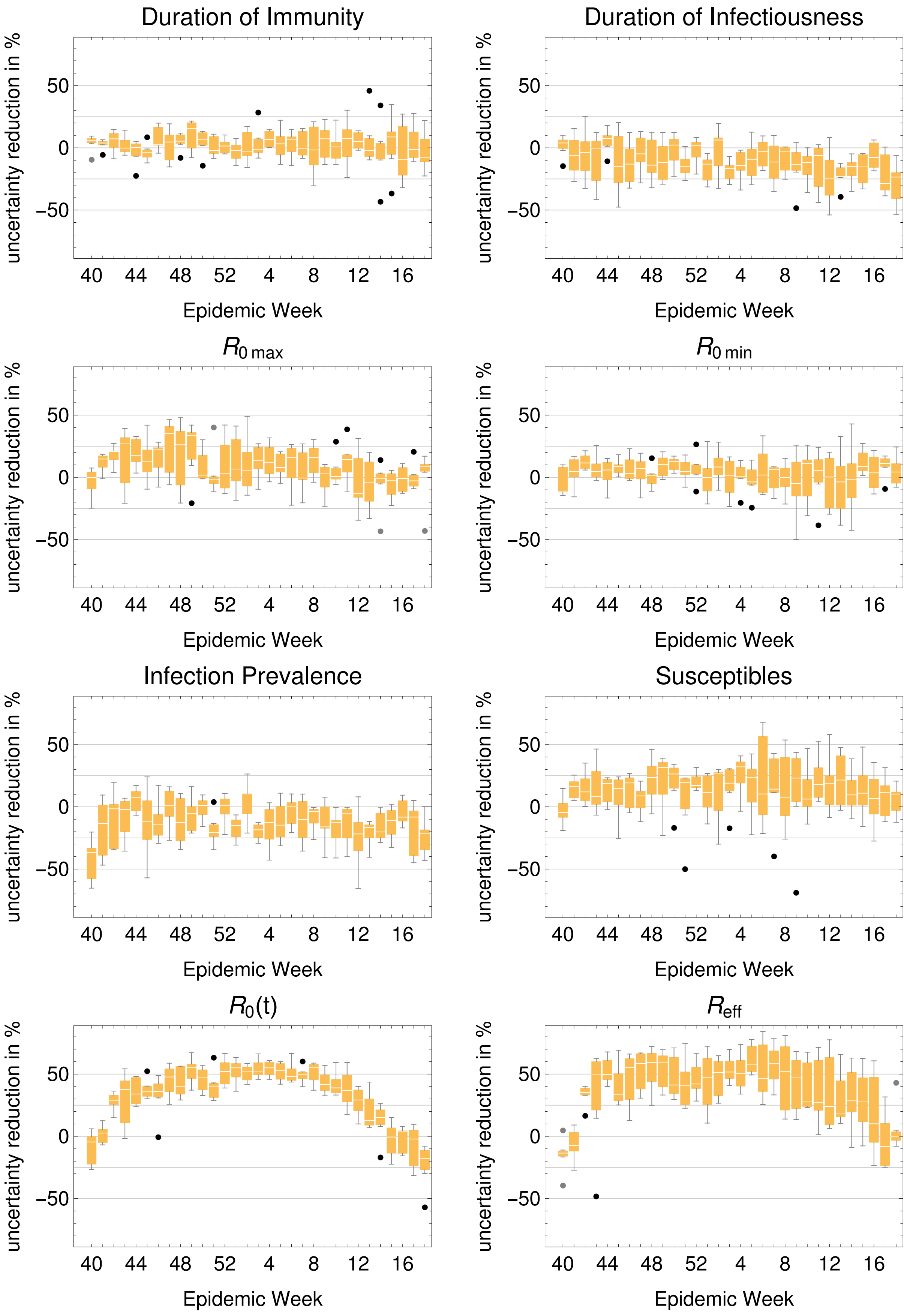
Duration of Immunity
Duration of Infectiousness
$R_{0\,max}$
$R_{0\,min}$
Infection Prevalence
Susceptibles
$R_0(t)$
R_{eff}
uncertainty reduction in %
Epidemic Week
50
0
−50
40
44
48
52
4
8
12
16
