# Supplementary material for: Use of daily Internet search query data improves real-time projections of influenza epidemics
Source: J R Soc Interface. 2018 Oct 10;15(147):20180220. doi: 10.1098/rsif.2018.0220 (PMC6228485; doi:10.1098/rsif.2018.0220)
Supplement: Computer code and results [file rsif20180220supp3.gz › Code-and-results/plots/wiki-matching-cdc.pdf]

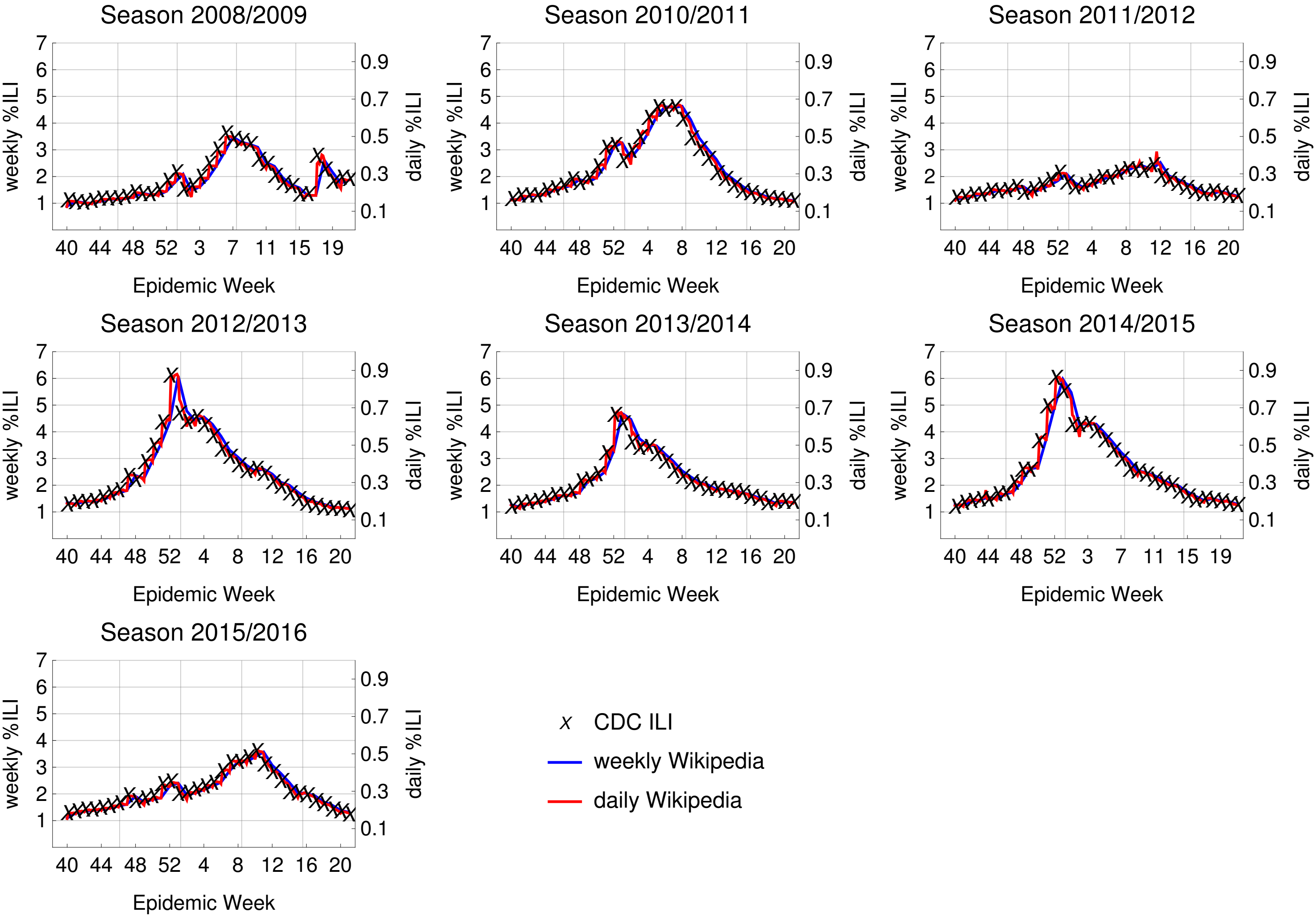
Season 2008/2009
Season 2010/2011
Season 2011/2012
Season 2012/2013
Season 2013/2014
Season 2014/2015
Season 2015/2016
weekly %ILI
daily %ILI
1 2 3 4 5 6 7
0.1 0.3 0.5 0.7 0.9
40 44 48 52 3 7 11 15 19
40 44 48 52 4 8 12 16 20
Epidemic Week
X CDC ILI
weekly Wikipedia
daily Wikipedia
