# Supplementary material for: Use of daily Internet search query data improves real-time projections of influenza epidemics
Source: J R Soc Interface. 2018 Oct 10;15(147):20180220. doi: 10.1098/rsif.2018.0220 (PMC6228485; doi:10.1098/rsif.2018.0220)
Supplement: Computer code and results [file rsif20180220supp3.gz › Code-and-results/plots/wiki-matching-cdc2008.pdf]

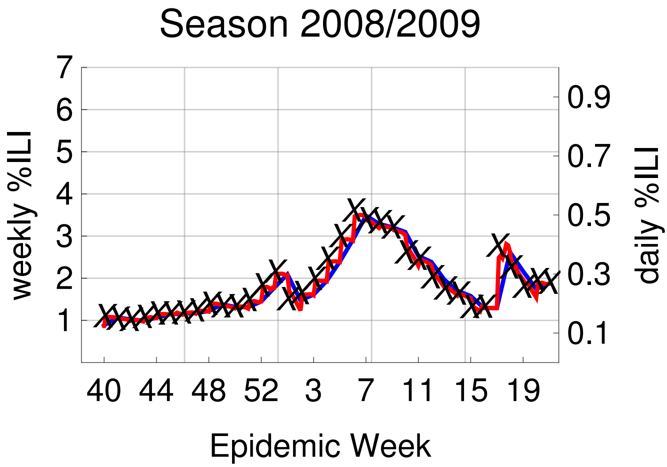

Season 2008/2009
weekly %ILI
daily %ILI
Epidemic Week
1
2
3
4
5
6
7
0.1
0.3
0.5
0.7
0.9
40
44
48
52
3
7
11
15
19
