# Supplementary material for: Use of daily Internet search query data improves real-time projections of influenza epidemics
Source: J R Soc Interface. 2018 Oct 10;15(147):20180220. doi: 10.1098/rsif.2018.0220 (PMC6228485; doi:10.1098/rsif.2018.0220)
Supplement: Computer code and results [file rsif20180220supp3.gz › Code-and-results/plots/SI-confid-plots.pdf]

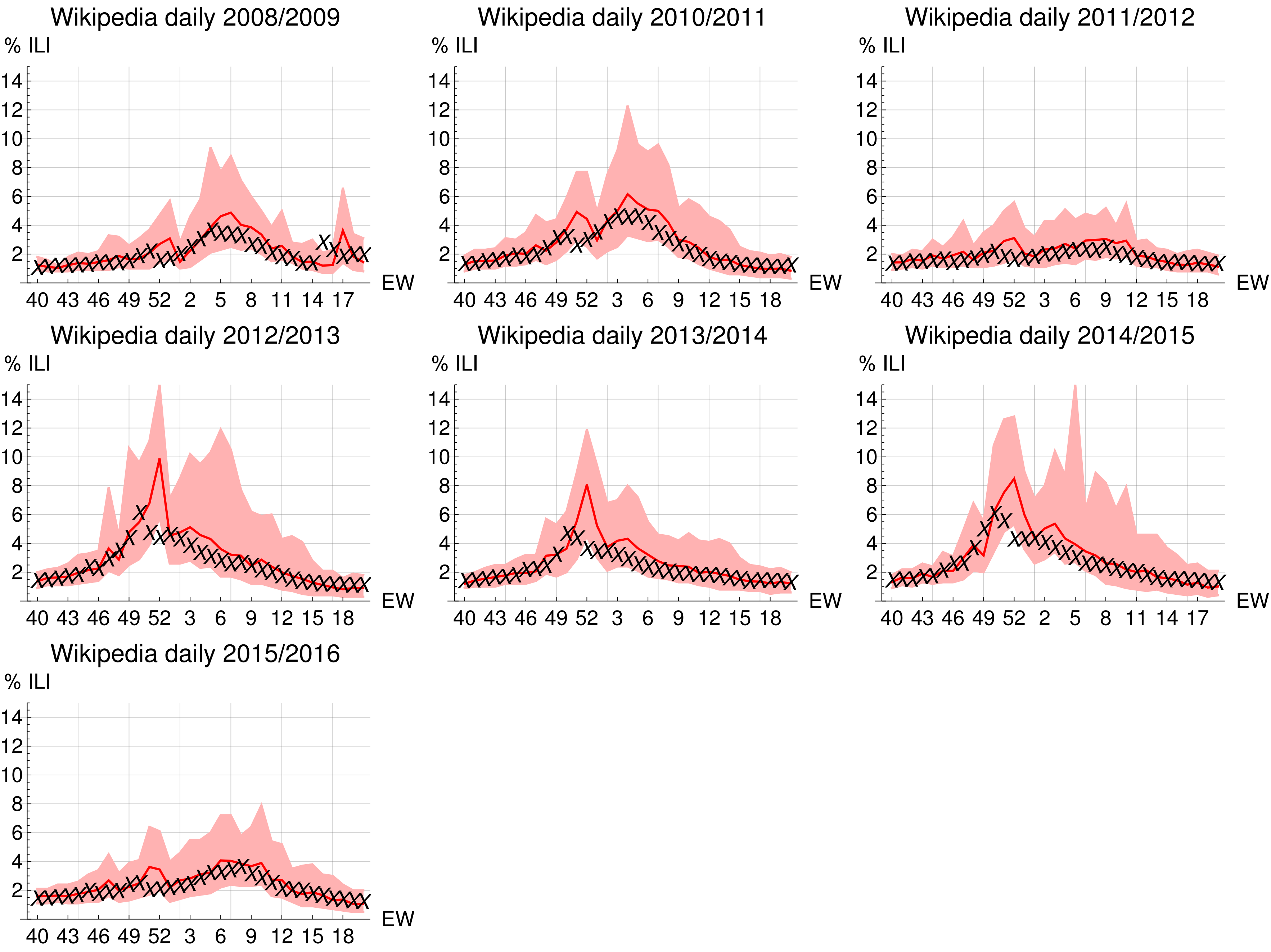
Wikipedia daily 2008/2009
% ILI
2 4 6 8 10 12 14
EW
40 43 46 49 52 2 5 8 11 14 17
Wikipedia daily 2010/2011
% ILI
2 4 6 8 10 12 14
EW
40 43 46 49 52 3 6 9 12 15 18
Wikipedia daily 2011/2012
% ILI
2 4 6 8 10 12 14
EW
40 43 46 49 52 3 6 9 12 15 18
Wikipedia daily 2012/2013
% ILI
2 4 6 8 10 12 14
EW
40 43 46 49 52 3 6 9 12 15 18
Wikipedia daily 2013/2014
% ILI
2 4 6 8 10 12 14
EW
40 43 46 49 52 3 6 9 12 15 18
Wikipedia daily 2014/2015
% ILI
2 4 6 8 10 12 14
EW
40 43 46 49 52 2 5 8 11 14 17
Wikipedia daily 2015/2016
% ILI
2 4 6 8 10 12 14
EW
40 43 46 49 52 3 6 9 12 15 18
